# Supplementary material for: Statistical mechanics for metabolic networks during steady state growth
Source: Nat Commun. 2018 Jul 30;9:2988. doi: 10.1038/s41467-018-05417-9 (PMC6065372; doi:10.1038/s41467-018-05417-9)
Supplement: Supplementary file 1 — Supplementary Information [file 41467_2018_5417_MOESM1_ESM.pdf]

Supplementary Information
Statistical mechanics for metabolic networks during steady-state growth

Supplementary Methods

The sampling algorithm

We sampled the space of steady states of the model using a hit-and-run Markov Chain Monte Carlo algorithm, subject to an ellipsoidal preprocessing step in order to tackle ill-conditioning. The uniform sampling of convex bodies by means of Monte Carlo methods has been considered a breakthrough in computational convex analysis, e.g., it makes feasible the calculation of the volume which is otherwise a computationally difficult problem [1]. A fast and popular algorithm to sample points inside convex bodies is the hit-and-run (HR) Markov Chain Monte Carlo [2, 3], which works along the following lines. Given a D-dimensional convex polytope $\mathcal{P}$, from which one wants to sample, and a point inside the polytope, $x_k \in \mathcal{P}$:

- Choose a uniformly distributed direction θ_k, that is, a point generated from the uniform distribution on the D-dimensional unit sphere. This can be done with the Marsaglia method, i.e., by generating D independent gaussian random variables with zero mean and unit variance, and then normalizing the vector to unit length.
- Generate t uniformly on the interval $[t_{\min}, t_{\max}]$, where $t_{\min}$ ($t_{\max}$) is the minimum (maximum) value of t such that $x_k + t\theta_k \in \mathcal{P}$;
- Update $x_{k+1} = x_k + t\theta_k$, start again.

The starting point can be found, for instance, by interpolating between two vertices obtained by linear programming. The second step requires finding the

intersections among a line and $\mathcal{P}$. In order to perform the HR dynamics we should always use a full-dimensional representation of the convex set. The mixing time of the HR, i.e., the time to converge to the desired distribution, scales as a polynomial of the dimensions of the body but the method can suffer from ill-conditioning; more precisely, the mixing time τ scales like [4]

$$\tau \sim O\left(D^2 \frac{R^2}{r^2}\right), \tag{1}$$

where R, r are the radii of respectively the minimum inscribing and the maximum inscribed balls. The prefactor R/r can be reduced to a polynomial of D by extracting θ_k from the surface of a matching ellipsoid instead of the unit sphere. The ellipsoid of maximum volume inscribed inside $\mathcal{P}$, i.e., the Loewner-John ellipsoid [5], would reduce the prefactor to $O(D)$, but the problem of finding it is NP-hard. Below we describe a method due to L. Lovasz [6] that finds in polynomial time an approximate matching ellipsoid that reduces the prefactor to $O(D^{3/2})$.

Preprocessing

We want to construct a couple of concentric ellipsoids E, E' matching the polytope $\mathcal{P}$, i.e., such that $E' \subseteq \mathcal{P} \subseteq E$, where E' is obtained from E shrinking by a factor $O(D^{3/2})$. This is called weak Loewner–John pair. We define a series of enclosing ellipsoids E_k, starting with E as the sphere with center in the origin and radius R large enough in order to inscribe the body, according to the following lines:

- INPUT: An ellipsoid E_k with its center x_k.
- Check if $x_k \in \mathcal{P}$, if yes go to 2, if no go to 1.
- 1) Consider a hyperplane separating x_k and $\mathcal{P}$, and the halfspace H enclosing $\mathcal{P}$, calculate the ellipsoid of minimal volume enclosing $H \bigcap E_k$; go to OUTPUT 1.
- 2) Determine the endpoints of the axis of E_k, shrink the ellipsoid and check if the shrunk ellipsoid E'_k is inside the body. If yes, go to OUTPUT 2, if no go to 3.
- 3) Consider the endpoint of an axis of the shrunk ellipsoid outside $\mathcal{P}$, e.g. x'_k; consider a hyperplane separating x'_k and $\mathcal{P}$, and the halfspace enclosing $\mathcal{P}$; calculate the ellipsoid of minimal volume enclosing $H \bigcap E'_k$; go to OUTPUT 1.
- OUTPUT 1: A new ellipsoid E_{k+1} of lower volume with center x_{k+1}, update k, repeat from INPUT.
- OUTPUT 2: A weak Loewner-John ellipsoid.

Upon calculating the reduction in volume of the enclosing ellipsoid after one step, it can be demonstrated that this series converges in polynomial time to a weak Loewner–John pair. We refer to [7] for demonstrations and formulae.

Source Code

We have provided in `doi:10.15479/AT:ISTA:62` a C++ code implementing the Lovasz preprocessing as well as the Hit-and-Run algorithm and the polytope representation of the metabolic network used in this study. Please refer to the `README.txt` file for further information.

Metabolic network and flux data

We considered the model of the *E. coli* catabolic core from the metabolic genome scale reconstruction iAF1260 [8]. Apart from mass-balance (purely stoichiometric) constraints, we have considered the bounds on reaction fluxes from the reversibility assignments provided within the model and default bounds on uptakes reflecting glucose limited aerobic conditions. We considered the dataset from [9] that includes extensive flux, enzyme, mRNA and metabolite level measurements of *E. coli* populations growing in steady-state in a glucose limited minimal medium in several conditions (dilution rates and/or gene knock outs). We considered further experimental estimates of the core carbon metabolism from the database [10], where we collected experiments [11, 12, 13, 14, 15, 16, 17, 18, 19, 20, 21, 22] on wild type *E. coli* performed in glucose limited medium and dilution rates not exceeding 0.5h^{-1}, i.e. below the acetate switch [23], red from which we single out experiments performed at growth rates of $\lambda = 0.1$ and 0.2h^{-1}, respectively.

Thermodynamics constraints implementation

Internal closed thermodynamically unfeasible flux cycles can arise in models upon incorrect handling of thermodynamic constraints [24] and, even if they have a neutral effect on the biomass, they can lead to an incorrect scaling analysis [25]. We have recurred with the default reversibility bounds provided with the model. In the model analyzed we have checked that no non-trivial thermodynamic inconsistencies are present using the methods defined in [26, 27]. Only one trivial internal cycle of length two has been detected between fumarate reductase (FRD7) and succinate dehydrogenase (SUCDi) whose prescribed bounds are thus thermodynamically inconsistent. This has been corrected by considering a lumped reversible reaction with the same stoichiometry.

The logistic growth model: further details

We report here for sake of completness i) the demonstration that the law of large numbers holds in the thermodynamic limit for the total number of cells in the logistic growth model, in turn validating asymptotically the mean field

approximation; ii) an approximate solution of the self consistent equation for β, giving further insights on its dependence upon the carrying capacity N_c and inoculum size N_0. The total number of cells N follows infact the equation

$$N = \sum^{N_0} e^{\beta\lambda_i} \tag{2}$$

where the λ_i are independent random variables identically distributed with the density $q(\lambda)$. Its average over disorder is thus (upto a factor) the moment generating function (Laplace transform) of $q(\lambda)$

$$\overline{N} = N_0 \int d\lambda q(\lambda) e^{\beta\lambda} \tag{3}$$

we have for the average of the square ($Z(\beta) = \int d\lambda q(\lambda) e^{\beta\lambda}$)

$$\overline{N^2} = N_0(N_0 - 1) Z^2(\beta) + N_0 Z(2\beta) \tag{4}$$

Upon considering the approximation $q(\lambda) = (a+1)(1-\lambda)^a$ and $\beta > a$ we can compute the relative fluctuations

$$\frac{\overline{(N-\overline{N})^2}}{(\overline{N})^2} \simeq \frac{1}{N_0}\left(\frac{\beta^{a+1}}{(a+1)!} - 1\right) \tag{5}$$

that vanishes in the aforementioned limit $N_0, K \to \infty$, $K/N_0 \to$ const., since β is finite and given asymptotically ($t \to \infty$) by the equation $Z(\beta) = K/N_0$. However such convergence can be very slow given the large prefactor and finite size corrections shall be worked out in future studies, possibly employing recent techniques developed in the the field of statistical mechanics of disordered systems [28]. Further $Z(\beta)$ can be approximately computed analytically giving

$$Z(\beta) = \frac{(a+1)e^{\beta}\gamma(a+1,\beta)}{\beta^{a+1}} = \frac{N_c}{N_0} \tag{6}$$

where $\gamma(a+1,\beta)$ is the lower incomplete Gamma function. For $\beta > a \gg 1$, we have approximately

$$\log\frac{N_C}{N_0} \simeq \beta - (a+1)(\log\frac{\beta}{a+1} + 1). \tag{7}$$

It can be seen that, for $\beta \gg a$ very large, if we consider an "equivalent" population growing at the maximum growth rate λ_{max}, β is the time (in units of λ_{max}^{-1}) where the carrying capacity and the steady state are reached $N_C \simeq N_0 e^{\beta\lambda_{max}}$ (upon restoring units). We report that the values of β calculated from this formula for a typical value in batch culture ($N_C/N_0 \simeq 10^6$) and extrapolated for the value in a mother machine ($N_C/N_0 \simeq 4$) as, respectively, $\beta \simeq 50$ and $\beta \simeq 20$ whereas we infer from experiments $\beta \simeq 100$ and $\beta \simeq 66$. Thus the simple logistic model underestimates β, but it correctly predicts that in batch cultures it is larger with respect to the mother machine, given the larger N_C/N_0.

Supplementary Notes

Supplementary Note 1

- *Figure 2A*
  Flux intensities have been normalized with respect to the glucose uptake (i.e. expressed in relative values). The flux values reported for the max entropy model refer to the value β^* that fixes the average growth rate to the experimental one along the following lines:
  - λ_{max} is inferred for each replicate from the reported experimental glucose uptake by means of flux balance analysis.
  - For each replicate the average growth rate is renormalized with respect to the maximum, $x = \frac{\langle\lambda\rangle}{\lambda_{max}}$. This is averaged over replicates to obtain $\bar{x}$.
  - The adimensional value $\beta^*\lambda_{max}$ that fixes the experimental value $\bar{x}$ is computed (this can be obtained analytically, see below Fig 3A,G).

  We considered two datasets at growth rate of, respectively, $\lambda = 0.1, 0.2\text{h}^{-1}$, (with sample size 12 and 7 sets of flux measurements) that we provide in .xls files. We obtain in both cases $\beta^*\lambda_{max} \approx 120$.

- *Figure 2B*
  We considered 10^2 values of β spanning the interval $\left[10^{-2}, 10^4\right]$ uniformly in logarithmic scale. For each value of β we sampled 10^5 flux configurations according to the max entropy distribution with the previously described hit-and-run Monte Carlo algorithm from which we calculate the estimates of the averages and variances. These are then compared to experimental values by computing the mean square error, $MSE = N_f^{-1}\sum_{i=1}^{N_f}\left(\langle v_i\rangle - V_i\right)^2$.

Supplementary Note 2

In the plots of figure 3 , fl ux in tensities ha ve be en no rmalized wi th re spect to the glucose uptake, while the growth rate has been rescaled by its maximum.

- *Figure 3A,G*
  The curves were calculated analytically along the following lines. We fit the marginal probability density of the growth rate, otained numerically by Monte Carlo sampling, with a Beta distribution, i.e. in relative units $x = \lambda/\lambda_{\text{max}}$:
  $$q(x) = (a+1)(1-x)^a, \tag{8}$$
  finding simply $a = D - 1 = 22$, where D is the dimension of the polytope, i.e. the growth rate is maximized in a subspace of dimension 0 (a vertex) [29]. The average growth rate as a function of β can be calculated

from the normalizing factor of the max entropy distribution,

$$Z(\beta) = \int_0^1 q(x) e^{\beta x} dx = \frac{(a+1)\gamma(a+1,\beta)e^{\beta}}{\beta^{a+1}}, \tag{9}$$

where γ is the lower incomplete gamma function and finally (Figure 3A)

$$\overline{x} = \frac{d \log Z}{d\beta}. \tag{10}$$

The minimal entropy reduction of the metabolic space upon fixing the average growth rate (Figure 3G) has been obtained by inverting the previous equation, $\beta = \beta(\overline{x})$, and performing a Legendre transform [30]

$$I(\overline{x}) \log 2 = \Delta S(\overline{x}) = \overline{x}\beta(\overline{x}) - \log Z(\beta(\overline{x})). \tag{11}$$

Both curves can be calculated numerically, the first directly, upon sampling the average growth rate at fixed β, while the second can be obtained in parametric form upon integration; in both cases we have found an excellent agreement with the analytical approximation.

- *Figure 3B-F*
  Averages, variances and correlations among fluxes have been calculated numerically by sampling max entropy distribution of the metabolic space with the previously described Monte Carlo method. We considered 10^2 values of β spanning the interval $\left[10^{-2}, 10^4\right]$ uniformly in logarithmic scale. For each value of β we sampled 10^5 flux configurations. Figure 3F has been obtained by numerical asymptotic analysis of flux correlations in the limit $\beta \to \infty$.

- *Figure 3H*
  We considered a simple model where K regulatory channels together provide the information, $I(\bar{\lambda})$, required to support a given average growth rate, $\bar{\lambda}$. Each channel is modeled as an additive Gaussian information channel whose capacity is given by $I_1 = \frac{1}{2}\log_2(1 + SNR)$, where SNR is the ratio of the signal to noise variance. To provide sufficient regulatory information we must have $I(\bar{\lambda}) \approx K I_1$. We assume that the variance of the signal is proportional to its mean, $\bar{S}$, so that $SNR = \alpha_1 \bar{S}$, for some constant α_1; this is approximately true in the simplest models of biochemical reaction pathways where the dominant source of noise is the shot (Poisson) noise due to finite number of signaling molecules. We further assume that the metabolic cost of regulation per channel is also proportional to the number of signaling molecules used, i.e., to the mean signal in the channel, and thus the total cost (written as the decrease in the growth rate) is $\Delta\lambda = -\alpha_2 K \bar{S}$. We can eliminate $\bar{S}$ by writing it out in terms of SNR, which can in turn be expressed in terms of $I(\bar{\lambda})$, to yield an expression for the effective growth rate that takes into account the cost of regulation:

$$\lambda_{\text{eff}}(\beta) = \bar{\lambda}(\beta) - \alpha K \left(2^{2I(\beta)/K} - 1\right), \tag{12}$$

Step	sample size	$\overline{\lambda}$ [min^{-1}]	$\lambda_{e,\max}$ [min^{-1}]	$\lambda_{\max}$ [min^{-1}]	$\beta\lambda_{\max}$ [adim.]
1	5204	0.022	0.029	0.034	66
2	3300	0.017	0.024	0.029	54
3	2252	0.012	0.018	0.023	45
4	1464	0.0085	0.013	0.017	45
5	1536	0.007	0.011	0.015	42

Supp.Table 1: Average growth rate, empirical maximum, inferred maximum and level of optimization, $\beta\lambda_{\max}$, from growth rate data.

where $\alpha = \alpha_2/\alpha_1$ is a constant proportional to the metabolic cost of signaling molecules.

Supplementary Note 3

In figure 4 the growth rate data have been obtained by analyzing images from a microfluidics device (mother machine) setting with 30 channels each containing 3 *E. coli* cells growing in steady-state in glucose-limited conditions. Their growth is monitored under increasing exposure to antibiotics (five concentration steps of tetracycline) specific for ribosome translation inhibition for 5h. Experiments have been performed on 3 strains; 3 technical repeats were carried out. Strains differed only by the fluorescent tags whose effects are not expected to have major contributions to the growth rates.

- *Figure 4A*
  The curve has been calculated analytically as described for Figure 3.

- *Figure 4C*
  The distributions have been obtained as follows. We disregard measurements during the first 15 time frames from each antibiotic step in order to obtain approximately stationary distributions; we also rejected outliers exceeding 4σ. For each antibiotic step, we lump together growth rate measurements pertaining to different channels, time, cell order in the channel (mother-daugthers), strains and repeats; this results in a sample size of $\simeq 1-5\cdot 10^3$ per antibiotic step. We performed a best fit (χ^2 minimization) of max entropy marginal growth rate distributions in the analytical approximation outlined in discussion for Figure 3 (above), and we retrieved the values for the two parameters β and $\lambda_{\max}$ that we report in Table 1, alongside of the average and empirical maximum of the growth rate.

- *Figure 4D*:
  Each data point stands for a distribution obtained upon lumping growth rate data measured in different channels (30) and time points ($\simeq 3$), i.e. it constitutes a unique sample whose size is $O(10^2)$. Each data point refers thus to a different antibiotic step (5), to a different cell ordering in

the channel (3), strain (3) and repeat (3) for a total of 135 points. We considered the usual estimators for the average and variance, while we have reported in all cases the empirical maximum for the growth rate.

Supplementary Note 4

Determining β using χ^2 fitting to flux measurements directly
In the maximum entropy approach the parameter β in Eq (3) of the main paper plays the role of the Lagrange multiplier that has to be set so that predicted average growth rate equals the measured growth rate (as in Eq (4) of the main paper). In this approach, described in the main paper, all average metabolic fluxes computable from the maximum entropy model after fixing β are predictions, to be compared to data.

We can, however, also treat the maximum entropy distribution of Eq (3) as a phenomenological fitting relationship suggested by theory, with a single free fitting parameter β to be set such that the model reproduces the fluxes directly as well as possible. In this case, we can also try to pool over flux measurements taken at different average growth rates and ask whether we obtain a better prediction than that of the FBA. This is shown in Supp.Fig 1A, where we fitted our model to 35 flux measurement experiments taken over the whole range of growth rates below the acetate switch, $\lambda \leq 0.5\text{h}^{-1}$.

Specifically, we defined as a quality-of-fit measure

$$\chi^2 = N_f^{-1} \sum_{i=1}^{N_f} \left(\langle v_i \rangle - V_i \right)^2 / \left(\sigma_i^2 + E_i^2 \right) , \tag{13}$$

where V_i is the measured flux (relative to glucose uptake) and E_i^2 the associated measurement error variance, while $\langle v_i \rangle$ and σ_i^2 are the mean (and variance, respectively) of the corresponding flux computed in the maximum entropy model. We then looked for the value β^* that directly minimized χ^2. The value of this at the optimal parameter β was significantly lower than in either the FBA or uniform limits, and this remained robustly true even with alternative goodness-of-fit measures (e.g., mean-squared-error, MSE$=N_f^{-1} \sum_{i=1}^{N_f} \left(\langle v_i \rangle - V_i \right)^2$ that does not normalize by a variance, σ_i^2, which grows as β is decreased). This is shown in Supp.Fig 1B. Predictions of similar quality were also obtained on three technical replicates of flux measurements shown in Supp.Fig 1C.

We simulated an enzyme knock out by removing the corresponding reaction from the model (one equality constraint). This leads to a new metabolic space that has been analyzed along the same lines as the unperturbed one. Flux estimates for knockouts from [9] consist of one single experiment with no repeats. The MSE improvement is the fractional difference between the minimum value for MSA and the value retrieved by FBA.

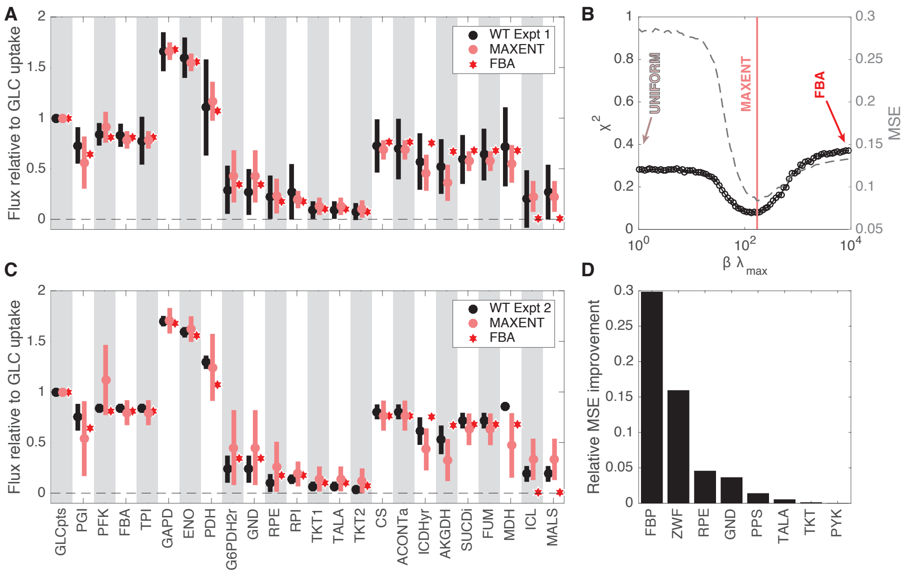

Supp. Figure. 1: **Direct fit of maximum entropy functional form, by χ^2 minimization outperforms FBA predictions in *E. coli.* (A, C)** Comparison of measured fluxes (black, mean $\pm$ SD over biological replicates; normalized to glucose uptake) with predictions of FBA (red stars) and of the maximum entropy model (pink, mean $\pm$ SD from the predicted joint distribution over fluxes). Data for (A) are a collection of 35 experiments from Ref [10]; data for (C) are three replicates from Ref [9]. Wild type *E. coli* was grown in glucose-limited medium with low dilution/growth rates (below 0.5h^{-1}, no acetate excretion). **(B)** Goodness-of-fit between flux measurements in (A) and the maximum en-tropy predictions, as a function of dimensionless $\beta\lambda_{\text{max}}$ parameter. χ^2 (left axis, black) weights the (squared) error in each flux prediction by the sum of measured experimental variance and the maximum entropy predicted variance (see text); MSE (right axis, gray) is an unweighted mean squared error between measurements and predictions. In both measures, the maximum entropy model (pink line at an intermediate value of $\beta^*\lambda_{\text{max}} \approx 170$) provides a better fit than the uniform ($\beta = 0$) or FBA ($\beta \to \infty$) limits. **(D)** Improvement in goodness-of-fit when using maximum entropy model over FBA for flux predictions in 7 *E. coli* mutant strains deleted for metabolic enzymes indicated on the horizontal axis; data from Ref [9].

Supplementary References

[1] Martin E. Dyer and Alan M. Frieze. On the complexity of computing the volume of a polyhedron. *SIAM Journal on Computing*, 17(5):967–974, 1988.

[2] V Turcin. On the computation of multidimensional integrals by the Monte

Carlo method. *Th Probab Appl*, 16:720–724, 1971.

[3] Robert Smith. The hit-and-run sampler: a globally reaching Markov chain sampler for generating arbitrary multivariate distributions. *Proc 1996 Winter Simul Conf*, pages 260–264, 1996.

[4] László Lovász. Hit-and-run mixes fast. *Math Program*, 86(3):443–461, 1999.

[5] Keith Ball. An elementary introduction to modern convex geometry. *Flavors of geometry*, 31:1–58, 1997.

[6] László Lovász. *An algorithmic theory of numbers, graphs and convexity*, volume 50. SIAM, 1987.

[7] Robert G Bland, Donald Goldfarb, and Michael J Todd. The ellipsoid method: A survey. *Operations research*, 29(6):1039–1091, 1981.

[8] Jeffrey D Orth, Tom M Conrad, Jessica Na, Joshua A Lerman, Hojung Nam, Adam M Feist, and Bernhard Ø Palsson. A comprehensive genome-scale reconstruction of escherichia coli metabolism—2011. *Molecular systems biology*, 7(1):535, 2011.

[9] Nobuyoshi Ishii, Kenji Nakahigashi, Tomoya Baba, Martin Robert, Tomoyoshi Soga, Akio Kanai, Takashi Hirasawa, Miki Naba, Kenta Hirai, Aminul Hoque, et al. Multiple high-throughput analyses monitor the response of e. coli to perturbations. *Science*, 316(5824):593–597, 2007.

[10] Zhengdong Zhang, Tie Shen, Bin Rui, Wenwei Zhou, Xiangfei Zhou, Chuanyu Shang, Chenwei Xin, Xiaoguang Liu, Gang Li, Jiansi Jiang, et al. Cecafdb: a curated database for the documentation, visualization and comparative analysis of central carbon metabolic flux distributions explored by 13c-fluxomics. *Nucleic acids research*, page gku1137, 2014.

[11] William R Farmer and James C Liao. Reduction of aerobic acetate production by escherichia coli. *Applied and environmental microbiology*, 63(8):3205–3210, 1997.

[12] K Al Zaid Siddiquee, MJ Arauzo-Bravo, and K1 Shimizu. Metabolic flux analysis of pykf gene knockout escherichia coli based on 13c-labeling experiments together with measurements of enzyme activities and intracellular metabolite concentrations. *Applied Microbiology and Biotechnology*, 63(4):407–417, 2004.

[13] Jochen Schaub, Klaus Mauch, and Matthias Reuss. Metabolic flux analysis in escherichia coli by integrating isotopic dynamic and isotopic stationary 13c labeling data. *Biotechnology and bioengineering*, 99(5):1170–1185, 2008.

[14] Lifeng Peng, Marcos J Arauzo-Bravo, and Kazuyuki Shimizu. Metabolic flux analysis for a ppc mutant escherichia coli based on 13c-labelling experiments together with enzyme activity assays and intracellular metabolite measurements. *FEMS microbiology letters*, 235(1):17–23, 2004.

[15] Marcel Emmerling, Michael Dauner, Aaron Ponti, Jocelyne Fiaux, Michel Hochuli, Thomas Szyperski, Kurt Wüthrich, JE Bailey, and Uwe Sauer. Metabolic flux responses to pyruvate kinase knockout in escherichia coli. *Journal of bacteriology*, 184(1):152–164, 2002.

[16] Qiang Hua, Chen Yang, Tomoya Baba, Hirotada Mori, and Kazuyuki Shimizu. Responses of the central metabolism in escherichia coli to phosphoglucose isomerase and glucose-6-phosphate dehydrogenase knockouts. *Journal of Bacteriology*, 185(24):7053–7067, 2003.

[17] Annik Nanchen, Alexander Schicker, Olga Revelles, and Uwe Sauer. Cyclic amp-dependent catabolite repression is the dominant control mechanism of metabolic fluxes under glucose limitation in escherichia coli. *Journal of bacteriology*, 190(7):2323–2330, 2008.

[18] Pablo I Nikel, Jiangfeng Zhu, Ka-Yiu San, Beatriz S Méndez, and George N Bennett. Metabolic flux analysis of escherichia coli creb and arca mutants reveals shared control of carbon catabolism under microaerobic growth conditions. *Journal of bacteriology*, 191(17):5538–5548, 2009.

[19] Mai Li, Pei Yee Ho, Shanjing Yao, and Kazuyuki Shimizu. Effect of lpda gene knockout on the metabolism in escherichia coli based on enzyme activities, intracellular metabolite concentrations and metabolic flux analysis by 13 c-labeling experiments. *Journal of biotechnology*, 122(2):254–266, 2006.

[20] Yoshihiro Toya, Nobuyoshi Ishii, Takashi Hirasawa, Miki Naba, Kenta Hirai, Kaori Sugawara, Saori Igarashi, Kazuyuki Shimizu, Masaru Tomita, and Tomoyoshi Soga. Direct measurement of isotopomer of intracellular metabolites using capillary electrophoresis time-of-flight mass spectrometry for efficient metabolic flux analysis. *Journal of chromatography A*, 1159(1):134–141, 2007.

[21] T Zhu, C Phalakornkule, S Ghosh, IE Grossmann, RR Koepsel, MM Ataai, and MM Domach. A metabolic network analysis & nmr experiment design tool with user interface-driven model construction for depth-first search analysis. *Metabolic engineering*, 5(2):74–85, 2003.

[22] Nicola Zamboni, Sarah-Maria Fendt, Martin Rühl, and Uwe Sauer. 13c-based metabolic flux analysis. *Nature protocols*, 4(6):878–892, 2009.

[23] Markus Basan, Sheng Hui, Hiroyuki Okano, Zhongge Zhang, Yang Shen, James R Williamson, and Terence Hwa. Overflow metabolism in escherichia coli results from efficient proteome allocation. *Nature*, 528(7580):99–104, 2015.

[24] D De Martino. Thermodynamics of biochemical networks and duality theorems. *Phys. Rev. E*, 87:053108, 2013.

[25] Daniele De Martino. Scales and multimodal flux distributions in stationary metabolic network models via thermodynamics. *Physical Review E*, 95(6):062419, 2017.

[26] Daniele De Martino, Matteo Figliuzzi, Andrea De Martino, and Enzo Marinari. A scalable algorithm to explore the gibbs energy landscape of genome-scale metabolic networks. *PLoS Comput Biol*, 8(6):e1002562, Jan 2012.

[27] Daniele De Martino, Fabrizio Capuani, Matteo Mori, Andrea De Martino, and Enzo Marinari. Counting and correcting thermodynamically infeasible flux cycles in genome-scale metabolic networks. *Metabolites*, 3(4):946–966, 2013.

[28] Yan V Fyodorov, Anthony Perret, and Grégory Schehr. Large time zero temperature dynamics of the spherical p= 2-spin glass model of finite size. *Journal of Statistical Mechanics: Theory and Experiment*, 2015(11):P11017, 2015.

[29] Daniele De Martino and Davide Masoero. Asymptotic analysis of noisy fitness maximization, applied to metabolism and growth. *Journal of Statistical Mechanics: Theory and Experiment*, 2016(12):123502, 2016.

[30] Daniele De Martino, Fabrizio Capuani, and Andrea De Martino. Growth against entropy in bacterial metabolism: the phenotypic trade-off behind empirical growth rate distributions in e. coli. *Physical biology*, 13:036005, 2016.
