# Supplementary material for: Statistical mechanics for metabolic networks during steady state growth
Source: Nat Commun. 2018 Jul 30;9:2988. doi: 10.1038/s41467-018-05417-9 (PMC6065372; doi:10.1038/s41467-018-05417-9)
Supplement: Supplementary file 3 — Description of Additional Supplementary Files [file 41467_2018_5417_MOESM3_ESM.pdf]

Description of Additional Supplementary Files

File Name: Supplementary Data 1
Description:

Supplementary data 1: Experimental estimates of the metabolic fluxes for the carbon catabolic core of E.coli, wild type in glucose-limited aerobic conditions, at average growth rates $\lambda = 0.1$ h^{-1} (12 experiments, technical replicates) and $\lambda = 0.2$ h^{-1} (7 experiments, technical replicates) collected from the database www.cecafdb.org (Zhang et al. NAR (2014), doi: 10.1093/nar/gku1137)
